# Symposium summary: epigenetic inheritance—impact for biology and society 23–25 August 2023, Zürich, Switzerland

**DOI:** 10.1093/eep/dvae002

**Published:** 2024-02-24

**Authors:** Leonard C Steg, Ellen Jaspers, Anar Alshanbayeva, Rodrigo G Arzate-Meija, Maria A Dimitriu, Katharina Gapp, Lola M Kourouma, Kerem Uzel, Isabelle M Mansuy

**Affiliations:** Laboratory of Neuroepigenetics, Brain Research Institute, Medical Faculty of the University of Zürich and Institute for Neuroscience, Department of Health Sciences and Technology of the ETH Zürich, Zürich 8057, Switzerland; Zürich Neuroscience Center, ETH and University of Zürich, Zürich 8057, Switzerland; Laboratory of Neuroepigenetics, Brain Research Institute, Medical Faculty of the University of Zürich and Institute for Neuroscience, Department of Health Sciences and Technology of the ETH Zürich, Zürich 8057, Switzerland; Zürich Neuroscience Center, ETH and University of Zürich, Zürich 8057, Switzerland; Laboratory of Neuroepigenetics, Brain Research Institute, Medical Faculty of the University of Zürich and Institute for Neuroscience, Department of Health Sciences and Technology of the ETH Zürich, Zürich 8057, Switzerland; Zürich Neuroscience Center, ETH and University of Zürich, Zürich 8057, Switzerland; Laboratory of Neuroepigenetics, Brain Research Institute, Medical Faculty of the University of Zürich and Institute for Neuroscience, Department of Health Sciences and Technology of the ETH Zürich, Zürich 8057, Switzerland; Zürich Neuroscience Center, ETH and University of Zürich, Zürich 8057, Switzerland; Laboratory of Neuroepigenetics, Brain Research Institute, Medical Faculty of the University of Zürich and Institute for Neuroscience, Department of Health Sciences and Technology of the ETH Zürich, Zürich 8057, Switzerland; Zürich Neuroscience Center, ETH and University of Zürich, Zürich 8057, Switzerland; Zürich Neuroscience Center, ETH and University of Zürich, Zürich 8057, Switzerland; Laboratory of Epigenetics and Neuroendocrinology, Institute for Neuroscience, Department of Health Science Technology, ETH Zürich, Zürich 8057, Switzerland; Laboratory of Neuroepigenetics, Brain Research Institute, Medical Faculty of the University of Zürich and Institute for Neuroscience, Department of Health Sciences and Technology of the ETH Zürich, Zürich 8057, Switzerland; Zürich Neuroscience Center, ETH and University of Zürich, Zürich 8057, Switzerland; Laboratory of Neuroepigenetics, Brain Research Institute, Medical Faculty of the University of Zürich and Institute for Neuroscience, Department of Health Sciences and Technology of the ETH Zürich, Zürich 8057, Switzerland; Zürich Neuroscience Center, ETH and University of Zürich, Zürich 8057, Switzerland; Laboratory of Neuroepigenetics, Brain Research Institute, Medical Faculty of the University of Zürich and Institute for Neuroscience, Department of Health Sciences and Technology of the ETH Zürich, Zürich 8057, Switzerland; Zürich Neuroscience Center, ETH and University of Zürich, Zürich 8057, Switzerland

**Keywords:** epigenetic inheritance, epigenetics, meeting report, transgenerational inheritance

## Abstract

The possibility that acquired traits can be transmitted across generations has been the subject of intense research in the past decades. This biological process is of major interest to many scientists and has profound implications for biology and society but has complex mechanisms and is therefore challenging to study. Because it involves factors independent from the DNA sequence, this form of heredity is classically referred to as epigenetic inheritance. Many studies have examined how life experiences and various environmental factors can cause phenotypes that are heritable and be manifested in subsequent generations. Recognizing the major importance and complexity of this research, the fourth edition of the Epigenetic Inheritance Symposium Zürich brought together experts from diverse disciplines to address current questions in the field of epigenetic inheritance and present recent findings. The symposium had sessions dedicated to epidemiological evidence and animal models, transmission mechanisms, methodologies and the far-reaching impact on society and evolution. This report summarizes the talks of speakers and describes additional activities offered during the symposium including poster sessions and an art competition on the topic of epigenetic inheritance.

## Introduction

Over the past two decades, epigenetic inheritance has been increasingly recognized as an important biological process that refers to the transmission of acquired traits. Its underlying mechanisms and its implications for health, disease and human evolution are areas of intense research. Animal and cellular models together with continued methodological advances to characterize, quantify and manipulate the epigenome have been essential to gain understanding of this biological phenomenon.

To foster the field’s expansion and facilitate collaborations, the fourth edition of the Epigenetic Inheritance Symposium Zürich was held on 23–25 August 2023 and gathered researchers from around the globe. Similarly to previous editions in 2017 and 2019, the 2023 symposium was in person and allowed people to physically meet again in Zürich, Switzerland (the 2021 symposium was online). The program covered a broad range of environmental exposures eliciting inter- or transgenerational effects in diverse species from *Caenorhabditis elegans* and fish to mice and humans (Session 1). A full day was dedicated to recent findings on the mechanisms of epigenetic inheritance (Session 2) and then half day to novel methodologies applicable to the field (Session 3) followed by a discussion about the interplay between epigenetic inheritance and evolutionary processes (Session 4). The symposium had 18 speakers and 135 participants and provided a dynamic platform for scientific discussions and exchanges. Further to science, the symposium continued its tradition to fuse science and art and offered an art exhibition on the theme of epigenetic inheritance. It featured original pieces created by participants, and the best piece was rewarded with a prize.

This report is a summary of the symposium reflecting major topics discussed. It does not include references associated with individual topics or speakers. Readers interested in bibliography are referred to PubMed or to each speaker’s respective research page. Some of the talks described unpublished data and are therefore summarized more briefly.

## Session 1: epidemiological evidence and animal models

The first session of the symposium was dedicated to recent evidence for the transmission of acquired traits in humans and animals. The speakers presented a range of environmental exposures that can induce inter- or transgenerational effects and highlighted potential therapeutic strategies.

The session started with a talk by Susan Ozanne (University of Cambridge, UK) on the impact of *in utero* exposure on long-term cardiometabolic health. In the past three decades, epidemiological studies uncovered a link between birth weight and the long-term risk of adult-onset diseases like Type 2 diabetes and cardiovascular diseases. This introduced the concept of “Developmental Origins of Health and Disease” (DoHAD), the enduring impact of the environment in early development, including *in utero* factors, on future health outcomes. The effects of nutrition on physiology and health in exposed individuals and their offspring were discussed, along with different animal models of nutritional insults. These models involved low protein diet, caloric restriction or maternal obesity and were shown to cause similar phenotypes in the offspring with three key features: (i) permanent structural changes in different organs, most likely due to inadequate level of essential hormones or nutrients during critical periods of development, (ii) accelerated cellular aging of metabolic and reproductive tissues likely due to increased oxidative stress that can lead to telomere shortening and (iii) changes in epigenetic programming that can modify gene expression in affected tissues. Future challenges will be to demonstrate causality of individual effects, define tissue-specificity and understand age-related cardiometabolic health. A better understanding of the mechanisms of Developmental Origins of Health and Disease may help identify disease risk markers and allow the design of effective interventions to enhance the well-being of women and their children.

The second talk was by Andrew Feinberg (Johns Hopkins University, USA), who discussed how the integration of genetics, epigenetics and environment and the contribution of these factors to the mechanisms of neuropsychiatric diseases can be studied in humans and animal models. The Feinberg lab has analyzed DNA methylation (DNAme) in sperm, together with social responsiveness scale scores of the respective fathers and their children. These analyses identified an association between paternal germline methylation and autistic traits in the 3-year-old offspring. This emphasized the potential contribution of sperm epigenetic signatures to autism, especially in families with a history of autism spectrum disorder. Switching to animal models, the lab also investigated three different mouse strains (C57BL/6 J, A/J and NOD/ShiLtJ) and their respective response to a high-fat diet. The diet led to varying degrees of metabolic syndrome in different mouse strains. There were both overlapping and distinct changes in gene expression and DNAme associated with the metabolic phenotypes. These findings suggest genotype-specific effects of diet that can allow genotype-adjusted therapeutic interventions as demonstrated in a pilot study using a metabolic drug, GW4064, to target farnesoid X receptor pathways.

A short talk by Ramji Bhandari (University of North Carolina at Greensboro and University of Missouri, USA) then described the partial reversal of transgenerational non-alcoholic fatty liver disease induced by ancestral bisphenol A (BPA) exposure in medaka fish. Non-alcoholic fatty liver disease symptoms due to BPA exposure persisted for five generations and could be transmitted by both, the male and female germline. Treatment with vitamin C during sensitive life stages was shown to reverse most BPA-induced differentially methylated regions (DMRs) and block non-alcoholic fatty liver disease development and progression. These results point to vitamin C as a potential nutritional agent to target BPA-induced liver disease.

Then, Ali Jawaid (Nencki Institute of Experimental Biology, Poland) discussed how childhood trauma, a high-risk factor for psychiatric and physical health conditions, can be associated with RNA alterations in humans. Assessment of small RNA in serum, sperm and milk obtained from human cohorts in Pakistan, Bosnia and Poland with individuals who experienced trauma identified micro-RNA signatures of trauma-related symptoms. Some of these micro-RNA changes were observed in all examined tissue types and were linked to cholesterol signaling. These results highlight the potential role of lipid metabolism in the long-term and intergenerational effects of trauma exposure, which is currently studied using mouse models and *ex vivo* approaches.

Grigorios Fanourgakis (Friedrich Miescher Institute, Switzerland) focused on the role of DNAme in shaping chromatin in male mouse germ cells and early embryos. The approach was to reduce the DNAme level in developing male germ cells by deleting *Dnmt3a* and *Dnmt3b* genes in undifferentiated spermatogonial cells. The mutant germ cells completed spermatogenesis and gave rise to sperm cells albeit with reduced DNAme across the genome. Reduced DNAme, especially at guanine-cytosine-rich loci, was accompanied by a gain of nucleosomes in sperm. A fraction of these hypomethylated sites also showed increased H3K4me3. Early two-cell stage embryos generated with hypomethylated sperm showed precocious establishment of H3K4me3 at paternal alleles. Most embryonic changes of H3K4me3 were not related to sperm-borne H3K4me3 changes. The results suggest that paternal DNAme contributes to the shaping of early embryonic chromatin and its modifications.

The session continued with Charlotte Cecil (Erasmus Medical Center, the Netherlands), who emphasized the value of collaborative efforts within consortia projects like the Pregnancy and Childhood Epigenetics and Horizon Europe FAMILY. The aim of these initiatives is to gain a deeper understanding of the risk factors for neuropsychiatric diseases in childhood and adolescence. The Cecil lab is exploiting large population-based birth cohorts to answer the question of why the risk of developing a condition significantly increases in the offspring when at least one parent is affected by a disease. The challenges in disentangling psychological, genetic and potential epigenetic transmission routes in humans, particularly in the context of mental health, underscore the importance of prospective longitudinal designs of upcoming studies. Preliminary results from the Pregnancy and Childhood Epigenetics consortium were presented, which included 39 prospective birth/child cohorts in which DNAme was examined in whole blood. The study attempted to integrate findings from all cohorts; however, challenges arose due to discrepancies between individual cohorts. Variance in clinical variables such as the age of disease onset or sample collection, as well as differences in sample processing methods, eventually resulted in small effect size in the reported DNAme changes. The results further highlighted the need for standardized sample collection and selection criteria, especially in studies investigating dynamic changes. The FAMILY project will additionally incorporate paternal effects, aiming to differentiate transmission routes beyond DNAme. The project will use a combination of population-based studies, high-risk offspring studies and animal experimental models. Finally, the use of trio studies that leverage genetic information from both parents was presented as a promising approach to uncover additional risk factors for neuropsychiatric diseases.

Bruce Blumberg (University of California, USA) shifted the focus back to the pressing global issue of obesity and associated health conditions. Drawing upon compelling data from various statistical observations in the USA, he underscored the consistent rise in obesity over the past five decades, a phenomenon not solely due to caloric intake or genetic factors. Instead, obesity seems to emerge in parallel to the unconscious exposure to a multitude of endocrine obesogenic disruptors in food, water, food packaging, personal care products and cleaning material. These obesogenic substances cause abnormal fat storage and adipogenesis, and over 50 such compounds have already been identified. Notably, these compounds are all legally authorized without considering their potentially additive and detrimental effects on disease risk and health outcomes. The complex interactions of tributyltin, an obesogenic endocrine disruptor, with nuclear receptors like retinoid X receptor were highlighted. These interactions lead to epigenetic changes that the Blumberg lab extensively studied in a transgenerational rodent model of prenatal exposure. The three main goals were to (i) understand the mechanisms of trait transmission across generations, (ii) identify changes in somatic tissues that facilitate fat accumulation in the context of diet and (iii) discover biomarkers of exposure. The results point to a transgenerational reconstruction of altered chromatin structure as a mechanism for transmission. B Blumberg concluded the talk with the hypothesis that large scale changes in chromatin structure rather than specific epimutations in individual genes propagate changes induced by prenatal exposure to tributyltin across multiple generations.

## Session 2: transmission mechanisms

The second session was devoted to the mechanisms of inter- or transgenerational epigenetic inheritance and discussed findings on various factors and processes that may be implicated including DNAme, non-coding RNAs, histone post-translational modifications (HPTMs), and 3D organization of the genome, their interplay and collective impact on germ and somatic cells.

The session started with Aurora Ruiz-Herrera (Autonomous University of Barcelona, Spain), who addressed two key topics in relation to 3D chromatin organization in male germ cells: (i) the organizational dynamics of 3D chromatin during male germ cell differentiation and (ii) the constraints governing 3D genome reorganization in germline development. To study changes in chromosome conformation during spermatogenesis, her lab developed a method to isolate germ cells at different differentiation stages in testis based on the cells’ DNA content and chromatin complexity. Using several methods including Hi-C, RNA-seq, ChIP-seq and microscopy, the lab reconstructed a high-resolution structural and functional atlas of male mouse germ cells during spermatogenesis. This comprehensive dataset was used to demonstrate how the interplay between genome-wide cohesion occupancy and transcriptional activity is shaping 3D remodeling during spermatogenesis. The impact of chromosomal fusions on higher-order chromatin organization was studied in a wild mouse strain (the Western European house mouse, *Mus musculus domesticus*), which can naturally harbor a Robertsonian fusion. This revealed that chromosomal fusion alters nuclear architecture in both meiotic and post-meiotic cells. These findings underscore that chromosomal fusion induces modifications of the chromosomal landscape of germ cells, potentially influencing the genome’s regulatory functions.

Next, Michael Skinner (Washington State University, USA) discussed the significance of incorporating generational toxicology in standardized toxicology studies. Among many toxic compounds tested in his lab, some have shown no effect in the exposed generation but major defects in subsequent generations that were not directly exposed. For instance, glyphosate, a widely used agricultural pesticide, had no evident impact on the health of F1 mice following direct exposure. However, both males and females from F2 and F3 generations exhibited obesity and females had ovarian diseases and parturition anomalies. Analyses of spermatogenic stages showed that epimutations are induced by toxicant exposure in a dynamic fashion and throughout all steps of spermatogenesis. Notably, a substantial proportion of DMRs emerged during testicular development, comprising ∼90% of total DMRs, while only ∼10% arose during epididymal transit for sperm maturation. A study on sperm epimutations after fertilization showed that a majority of sperm DMRs induced by toxicant exposure escape demethylation during early developmental stages, similar to imprinted genes. All these studies highlight the importance of studying transgenerational effects of toxic compounds, considering “hidden” effects in exposed animals that manifest only in the offspring. The talk was concluded by emphasizing the strength of multi-omics approaches combining analyses of multiple epigenetic modalities such as DNAme, HPTMs and non-coding RNAs.

A talk by Sarah Kimmins (McGill University, Canada) discussed the impact of environmental stressors on the sperm epigenome and their potential role in intergenerational inheritance and disease. It showed data supporting the idea that sperm chromatin can act as a sensor of environmental challenges. Dietary stress such as folate deficiency or high-fat diet can alter the distribution of H3K4me3 genome-wide in sperm. Changes in H3K4me3 occurred at gene promoters and potential enhancers related to embryonic development and metabolic processes. Remarkably, the pattern of H3K4me3 in sperm resembled those seen in two- and eight-cell embryos, suggesting instructive roles of sperm chromatin in embryonic H3K4me3 deposition. Regions with environmentally induced H3K4me3 changes in sperm exhibited corresponding changes in embryos, correlating with altered gene expression. Furthermore, the Kimmins lab investigated how the sperm epigenome can influence extra-embryonic tissues like placenta. Male mice exposed to a high-fat diet have H3K4me3 changes at regulatory elements of placenta-related genes, which correlated with altered placental gene expression and potential differences in cellular composition. The findings were extended to humans showing that exposure to dichlorodiphenyltrichloroethane in South African men correlated with changes in H3K4me3 at ∼2000 genomic regions in sperm. Genes with altered H3K4me3 due to dichlorodiphenyltrichloroethane exposure were expressed in embryos, suggesting that also human sperm H3K4me3 can influence HPTM deposition and gene expression in embryos. A model highlighting the role of sperm H3K4me3 as an environmental sensor was proposed. Environmental challenges lead to changes in H3K4me3 at promoters, enhancers and transposable elements of sperm cells (F0). The altered sperm chromatin then guides H3K4me3 deposition in embryos, resulting in the inheritance of environmentally driven sperm changes in the offspring (F1). These changes subsequently affect disease onset, potentially leading to further alterations in sperm chromatin and subsequent generations (F2).

In the following talk, Katharina Gapp (ETH Zürich, Switzerland) presented data supporting the role of sperm RNA in the intergenerational effects of stress exposure in mice. Strikingly, a single injection of dexamethasone (Dex) can induce changes in the composition of sperm RNA, similarly to that observed in models of chronic stress. The offspring derived from sperm from exposed males showed metabolic effects such as a compromised glucose metabolism and altered body mass index. Injection of sperm RNA from Dex-injected males into fertilized zygotes resulted in male and female offspring with differential sensitivity to stress and metabolic effects. To further investigate the mechanisms of intergenerational inheritance of Dex exposure, the lab examined chromatin accessibility of the sperm epigenome. Using a combination of Dex exposure and genetic manipulation, it could link changes in chromatin accessibility at components of glucocorticoid receptor signaling pathways and specific DNA modifications. Together, these results suggest a relationship between sperm chromatin accessibility through glucocorticoid receptor activity and DNAme dynamics in the context of Dex exposure.

Next, Jacquetta Trasler (McGill University, Canada) discussed perturbations in dietary folate and folate metabolism and their transgenerational effects. Folate metabolic cycle is an important source of methyl groups necessary for DNAme. By modifying folate intake throughout the lifetime of male mice, her lab found that both folate deficiency and a 20-fold folic acid supplementation resulted in decreased sperm count in the exposed generation and increased pre-weaning death in the offspring. Furthermore, both folate deficiency and supplementation induced sperm DNA hypomethylation in three consecutive generations. Although no significant overlap in the specific differentially methylated cytosines was observed across the three generations, the hypomethylated sites shared an enrichment for repetitive elements, especially young long interspersed nuclear elements in all generations. Using a different mouse model of folate deficiency through the genetic perturbation of a critical enzyme of folate metabolic cycle (5,10-methylenetetrahydrofolate reductase), the group observed sperm DNA hypomethylation in fathers and their progeny. Strikingly, hypomethylated regions were again enriched in young retrotransposons, suggesting their potential involvement in the transgenerational inheritance of epimutations.

Eric Greer (Washington University, USA) continued the session by discussing mechanisms of epigenetic inheritance in a model of parental starvation in *C. elegans*. The lab used radioactive methyl donors to track heritable changes in RNA methylation in subsequent generations. It observed a significant increase in 18S ribosomal RNA methylation that was passed to following generations. This heritable RNA methylation phenomenon was largely attributed to the methyltransferases DIMT-1 and BUD-23, while other ribosomal RNA methyltransferases did not seem to contribute to this transmission. Parental starvation caused intergenerational hormesis with increased resistance to heat stress and decreased fertility in the progeny. Alterations in the translation of genes associated with reproduction, longevity and stress responses were also observed. Strikingly, *dimt-1* and *bud-23* were required for the hormesis phenotype. Furthermore, *dimt-1* and *bud-23* knockdown resulted in translational alterations similar to those induced by starvation. These findings highlight the importance of ribosomal RNA methylation in mediating intergenerational effects of parental starvation in *C. elegans*.

Jamie Hackett (European Molecular Biology Laboratory, Italy) presented the work on intergenerational effects of a disrupted gut microbiome in mice. The microbiome can influence host physiology and is very sensitive to the environment. Research of the last decade showed a strong connection between the gut microbiome and bodily functions. Dysbiosis—an unbalanced gut microbiome—has been linked to a variety of pathologies, but its effect on offspring health remains unexplored. He discussed the potential for parental gut microbiome perturbations to influence parental reproductive systems and intergenerational offspring health.

Raffaele Teperino (Helmholtz Munich, Germany) presented data examining the role of spermatogenesis versus maturation during epididymal transit in the intergenerational inheritance of paternal overweight. He showed that overweight in mice reprograms the payload of sperm non-coding RNAs, with a pronounced impact on mitochondrial-derived small RNA. Importantly, using two independent human cohorts, clear evidence was provided that paternal overweight doubles the risk of obesity in the offspring and that mitochondrial-derived small RNAs in sperm are in significant linear association with BMI. The findings from his lab further highlight the potential of mitochondrial-derived small RNAs in mediating intergenerational inheritance.

The last speaker of the session, Yuta Takahashi (Altos Labs Institute of Science, USA), discussed recent work on the transgenerational inheritance of artificially induced DNAme and associated phenotypes. This study involved the generation of DNAme-edited mice through embryo microinjection of mouse embryonic stem cells with induced DNAme at two obesity-related loci. The edited mice sustained the induced DNAme and obesity phenotypes, which persisted across multiple generations (F2, F3 and F4) via both maternal and paternal lineage. The results showed that the edited DNAme was reduced in male and female primordial germ cells but reinstated in the epiblast. While the exact mechanism underlying this erasure and reestablishment remains unresolved, these findings provide causal evidence for transgenerational inheritance of acquired epigenetic marks and their associated phenotypes.

## Session 3: methodologies

The third session of the symposium focused on recent methodological advancements relevant for the field particularly 3D testicular organoid models and single-cell epigenomics.

Ina Dobrinski (University of Calgary, Canada) presented various models to study testicular development and function, including 2D co-culture systems of spermatogonial cells and supporting somatic cells, testicular tissue grafting and a recent 3D microwell testicular organoid model her lab developed. 3D testicular organoids more closely mimic *in vivo* responses of spermatogonia to retinoic acid stimulation than 2D cultures and have the potential to replicate testis-specific organization and its functions. The Dobrinski lab successfully cultured testicular organoids in several species including mouse, rat, pig, monkey and human. These models were used to replicate the effects of known reproductive toxicants like phthalic acid mono-2-ethylhexyl ester and cadmium chloride, which alter the transcriptome of testicular somatic cells and disrupt tight junction integrity. Organoid systems also proved versatile for studying soma-to-germline communication. The lab observed the transfer of extracellular vesicles from Sertoli cells to spermatogonia, a process that is being further studied in the organoid model. While *in vitro* spermatogenesis remains challenging in 3D testicular organoids, this limitation may be overcome by strategies aimed at enhancing somatic cell maturation. Continuous improvement of such complex *in vitro* models will undoubtedly help increase their potential for *in vitro* modeling of germ cells function in health and disease. The 3D organoid model can provide a powerful system to dissect soma-to-germline communication and the mechanisms of epigenetic inheritance.

Then Bing Ren (University of San Diego, California, USA) presented new advancements in single-cell epigenomics to characterize regulatory elements in gene networks and gain deeper understanding of mechanisms of health and disease. He introduced a modified single-cell Assay for Transposase-Accessible Chromatin using sequencing protocol utilizing combinatorial indexing, resulting in an atlas of over 1 million candidate *cis*-regulatory elements that are cell type-specific and detected across 222 human cell types. This resource helped determine the type of cells and specific genes linked to complex human traits and diseases caused by changes in non-coding DNA, showing important connections between cell types and diseases. An improved version of the previously published Paired-Tag strategy to profile HPTMs and gene expression in single cells was introduced. The recent modification—Droplet Paired-Tag—uses antibody-conjugated pA-Tn5 and the commercially available microfluidics 10×chromium single-cell multi-ome platform for faster and more accessible single-cell epigenome profiling. Droplet Paired-Tag was used to map cell type-specific candidate *cis*-regulatory elements and predict target genes of distal candidate *cis*-regulatory elements in adult mouse brain, where it had superior performance compared to other single-cell epigenomics techniques. The continuous advancements in high-throughput multi-omic profiling of single cells by the Ren lab and colleagues considerably expand research possibilities in gene regulation and epigenetics.

## Session 4: impact on society and evolution

The final session, initially planned to discuss the impact of epigenetic inheritance on society and evolution, had to be modified due to the cancellation of the two scheduled speakers shortly before the symposium. Fortunately, Carlos Guerrero-Bosagna (Uppsala University, Sweden) stepped in to present his research on the role of epigenetics in evolution. Through the introduction of the concepts of “Environmental Induction” and “Environmental Selection,” the talk outlined how evolution can potentially be driven by epigenetic factors. The significance of DNAme as a potential bridge between genetic and environmental factors in evolutionary processes was highlighted. This was illustrated by studies in birds, especially junglefowls, demonstrating that controlled environmental pressure, such as selection for tameness or specific behaviors, not only leads to phenotypic differences but also brings genetic and epigenetic variations. A methodological highlight was the introduction of Genotype-by-Sequencing and Methylated-DNA-Immunoprecipitation, an innovative approach that simultaneously studies genetic mutations and methylation changes. This approach not only reaffirmed the importance of DNAme in evolution but also revealed how specific genes are targeted during selection processes. Interestingly, while delving into the genetics of feather pecking behavior in chickens, it was shown that changes at the genetic, epigenetic and phenotypic levels are often non-overlapping, suggesting independent yet parallel paths of evolutionary pressure. Together, these results emphasized that environmental influences on genes, facilitated by mechanisms like DNAme, are equally vital drivers of evolution as genetic factors. The call for a “conceptual migration” of our understanding of evolution intends to challenge conventional beliefs and suggests that genetic and environmental elements may be more intertwined than previously thought.

The talk was concluded with a heartfelt tribute to Daniel Nätt, a respected colleague who recently passed away, and a reminder of his work and its lasting impact.

## Additional symposium activities

### Poster sessions and flash talks

In addition to oral presentations, 45 posters were presented during two dedicated sessions. Among these posters, nine presenters were chosen for short talks, which were presented in the afternoon of the first day. A poster prize was awarded at the end of the symposium. The winning poster “Transcriptome profiling of histone writers/erasers enzymes across spermatogenesis, mature sperm and pre-cleavage embryo: implications in paternal epigenome transitions and inheritance mechanisms” was presented by Candela Gonzáles (Instituto de Investigaciones Farmacológicas ININFA-UBA-CONICET, Buenos Aires, Argentina).

### Workshop

In the last afternoon of the symposium, a workshop was organized for registered participants and addressed five questions: (i) what are the best strategies to move toward assessing non-genetic inheritance in humans, (ii) what are the best animal models of inter- or transgenerational inheritance, (iii) which strategies can be used to distinguish genetic from epigenetic contributions to environmentally induced traits in the model, (iv) what are the most suitable cellular models to study the mechanisms of epigenetic inheritance and (v) whether and how to build an epigenome roadmap of the germline in relation to exposure and inheritance. These questions were shared with participants prior to the workshop, and a sixth question was added during the discussion: which societal goals and interventions should be considered once the mechanisms of inter- and transgenerational inheritance are revealed? The workshop was chaired by Katharina Gapp (ETH Zürich, Switzerland), Jamie Hackett (EMBL Rome, Italy) and Isabelle Mansuy (University and ETH Zürich, Switzerland) fostering vibrant and constructive discussions in an open and collegial atmosphere. A key takeaway from the workshop was a collective call for enhanced collaboration among researchers engaged in research on epigenetic inheritance. The proposition to establish a consortium dedicated to this field was put forward with the aim to advance research outcomes and enable collective efforts in engaging with funding agencies, lawmakers and—most importantly—the public, to achieve significant impact on both biology and society.

### Art exhibition and competition

Continuing the tradition of earlier symposia, art was present throughout the days and highlighted its natural connection with science. The symposium featured a small exhibition showcasing art pieces from previous symposia including “Waddington” by Florence Razoux in 2019 and “Transgenerational Memories” by Sandrine Donnio Renaud in 2020 and a novel creation—a rap song by French artist Meak (https://www.meak-highhood.com). In his song, Meak artfully elaborates on the generational trauma within families, set to emotive lyrics. The music video and translated lyrics were exhibited during the symposium, and the song was played to the audience.

**Figure 1:**
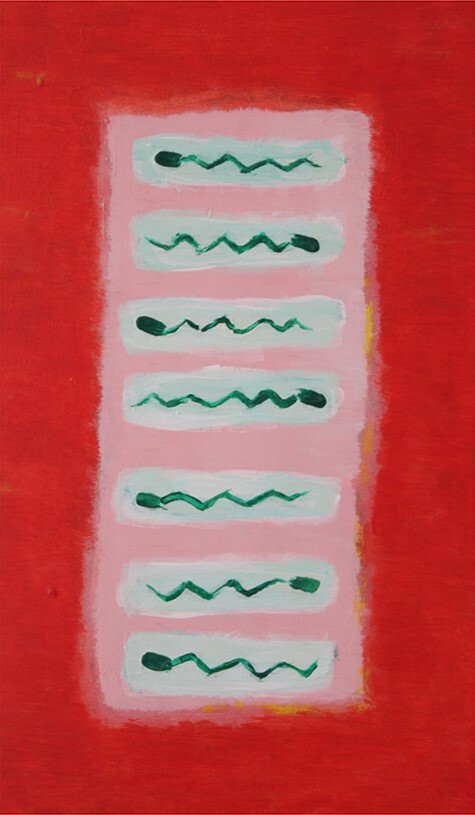
“Swimmers” by Signe Skog

An art competition was organized for all participants, resulting in four very nice and creative pieces. Among them, the acrylic painting titled “Swimmers” by Signe Skog (Linköping University, Sweden) was selected to be awarded (Fig. 1). These pieces reflected the inspiration of young researchers working on epigenetic inheritance and the artistic inspiration it stirs. We thank all talented artists who contributed to the exhibition and the competition and enriched our symposium with their creative perspectives and expressions.

## Data Availability

No data has been produced for this summary. For furhter details, refer to PubMed or to each of the speaker’s respective research pages.

